# Sparse estimation for structural variability

**DOI:** 10.1186/1748-7188-6-12

**Published:** 2011-04-19

**Authors:** Raghavendra Hosur, Rohit Singh, Bonnie Berger

**Affiliations:** 1 Computer Science and Artificial Intelligence Laboratory, MIT, Cambridge, MA, USA; 2 Dept. of Mathematics, MIT, Cambridge, MA, USA; 3 Dept. of Materials Science and Eng., MIT, Cambridge, MA, USA

## Abstract

**Background:**

Proteins are dynamic molecules that exhibit a wide range of motions; often these conformational changes are important for protein function. Determining biologically relevant conformational changes, or true variability, efficiently is challenging due to the noise present in structure data.

**Results:**

In this paper we present a novel approach to elucidate conformational variability in structures solved using X-ray crystallography. We first infer an ensemble to represent the experimental data and then formulate the identification of truly variable members of the ensemble (as opposed to those that vary only due to noise) as a sparse estimation problem. Our results indicate that the algorithm is able to accurately distinguish genuine conformational changes from variability due to noise. We validate our predictions for structures in the Protein Data Bank by comparing with NMR experiments, as well as on synthetic data. In addition to improved performance over existing methods, the algorithm is robust to the levels of noise present in real data. In the case of Human Ubiquitin-conjugating enzyme Ubc9, variability identified by the algorithm corresponds to functionally important residues implicated by mutagenesis experiments. Our algorithm is also general enough to be integrated into state-of-the-art software tools for structure-inference.

## Introduction

A central tenet of molecular biology is that a protein's three-dimensional (3D) structure is crucial to its function. Indeed the structural genomics initiative is producing an ever increasing number of structures at high resolution, providing accurate coordinates for each atom in the structure [1]. A protein's structure, however, is rarely static. Proteins are dynamic molecules, capable of exhibiting a wide range of motions and conformational variability [2,3]. Such conformational changes are important in biological functions such as enzymatic catalysis, cellular transport, and signaling [4,5]. It has been postulated that even subtle conformational changes may have important functional consequences [6].

A multi-conformer model, or ensemble, attempts to model variability by explaining the data using an ensemble of conformers, rather than just one conformer. Indeed, conformational variability in a protein might be present even in a single experiment, where the observed data is an average over multiple conformations [7,8]. Multi-conformer approaches have long been the norm when modeling NMR data. It has been suggested that, for an accurate representation of the physical heterogeneity in a protein, such multiple-conformer models also be used to explain X-ray crystallography data [8-10].

An open problem-- and the focus of this paper-- is understanding the nature of conformational variability implied by experimental data. The key challenge here is to distinguish variability resulting due to noise in experimental data and uncertainty in structure determination techniques from functionally relevant physical motion [9,11,12]. The problem is particularly difficult to solve with single-conformer approaches, given their limited ability to model the data. Indeed, this issue has been a driving force in the efforts toward ensemble approaches [8]. Even with the current ensemble approaches, it is difficult to disentangle a protein's physical motion (e.g. hinge or loop motions) from other kinds of protein motion (e.g., vibrational motion). The key problem is that limited sampling (i.e. number of conformations) and multiplicity of the problem make for weak statistical estimates [8,10,13]. While a growing number of tools address the problem of using ensembles to implicitly model conformational variability [7,10,12,14,15], they generally do not distinguish between variability due to noise vs. physical motion.

There have been some attempts to analyze structural variability, but using pairs of structures rather than ensembles. Conventional parameters such as torsional angle differences, temperature factors and root-mean-squared-distance (RMSD) values have been used to identify flexible regions. But they combine estimation noise and true variability into a single quantity; thus, they are of limited usefulness under noisy data (e.g., for low-to-medium resolution structures) (see Related Work, [11]). More importantly, conformational variability is best described over a population (i.e., ensemble) of conformations; pairwise comparison between structures implies such limited sampling of the conformational space that it may be unreliable for all but the least noisy datasets.

In this paper, we take a different approach to analyzing variability. Our approach is inspired by recent developments in regression-based predictive models in machine learning. The basic intuition behind the approach is to construct an ensemble of conformers that explain the experimental data and then use sparse estimation to distinguish between conformers that are just noisy versions of a base conformation (e.g., the PDB structure) and those that capture true conformational variability (relative to the base). Accordingly, structures sampled from a Gaussian distribution about the base structure should be more predictive of the base structure than structures displaying true variability. This allows us to separate out the biologically relevant variability due to physical motion using a feature selection technique, Lasso [16]. Lasso, which stands for "least absolute shrinkage and selection operator," is a regularized regression technique in which only the most significant predictor features are selected [16]. We illustrate the approach on X-ray crystallographic data, as it is the most common source of structural data. Our results demonstrate that the method compares favorably with previous approaches. It is more robust to specific parameter choices and produces fewer false positives and false negatives (see *Comparative Analysis*). In contrast to conventional approaches, we use Electron Density Maps (EDM), as opposed to 3-D coordinates used for pairwise structure comparison, for identification of true variability; this allows us greater power in accurately identifying true structural outliers without the need for any artificial parameters to model noise [17]. Finally, our predictions of true variable regions are in good agreement with the dynamics inferred from solution NMR experiments; the latter are presumably closer to the physical reality.

One of the key contributions of our work is in framing the problem as a sparse estimation problem, in a way that allows a wealth of machine learning knowledge to be applied to it. In particular, the problem of identifying sparse models that can be physically interpreted has recently gained much attention in machine learning, data mining and statistics due to the exponential growth in publicly available data [18]. We show here that identification of true variable regions in an ensemble is naturally formulated as a sparse learning problem via Lasso. This formulation allows us to rigorously deal both with noise in the experimental data and uncertainty associated with the structure-building process. Our approach of using Lasso is quite general, and can be applied to any structural data. Application of our method to proteins of interest may reveal interesting conformational changes that might go unnoticed due to the absence of alternate structural evidence, i.e., independently solved alternate conformations, which are still expensive and cumbersome to obtain.

A key intuition driving our approach is as follows: to identify true variability in a protein fragment, rather than performing a per-atom statistical test, we perform a whole-model statistical test. A per-atom test will essentially ignore correlated motions (even if small) between neighboring atoms; in contrast, a whole-model test will be able to identify even small correlated motions. We formalize this approach using the Lasso-based test. We exploit the idea of borrowing information from all the samples to make a reliable statistical inference on a particular sample. In contrast, a pairwise *t*-*statistic *approach uses information from only a single sample to make a decision [13].

### Related Work

Coordinate-based methods using pairwise comparisons have had reasonable success in identifying flexible regions [11,19]. However these techniques were designed to identify true flexibility in conformations that have been solved independently, where there is already some evidence of variability. Nigham et al. give a statistical test based on pairwise RMSD to identify regions showing true variability in the presence of noise. Key to their method is the assumption of a uniform, normal independent noise (artificially added) at each coordinate. However, this assumption typically does not hold in reality [20].

Related approaches rely on the use of various parameters such as torsion angle differences, temperature factors and RMSD. Torsion angle differences are highly sensitive to noise: small deviations in coordinates might cause significant changes in torsion angles [11]. Temperature factors (B-factors) are parameters used to model uncertainty in atomic positions; the value of the B-factor corresponding to an atom represents the degree of uncertainty in that atom's location in the model. This distribution accounts for small vibrations about an atom's position. However, B-factors tend to encapsulate in one value the conformational variability, as well as ambiguities related to inadequacies in data (e.g., related to crystal imperfections, errors in measurement of intensities). This problem is aggravated at medium-to-low resolutions (> 1.5*Å*). At such resolutions, B-factors act as "error-sinks," absorbing any errors (not necessarily related to protein motion) in the optimization and model building process [17].

A number of methods have been proposed to model multiple conformations that might give rise to X-ray crystallographic data from a single crystal [7,9,10,12]. Although independently optimized multi-conformer representations prove to be a very attractive solution, interpretation of what the ensemble represents is a gray area [9,12]. Knight et al. (2008) give a simple residue-level heuristic test based on the variance in the ensemble to identify true variability. However, there is no consensus method to identify true structural variability, and the interpretation of such ensembles is still the subject of debate [9,12].

## Results

**Figure 1:**
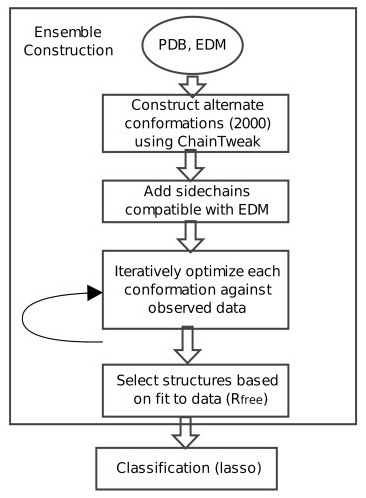
**Overview of the ensemble generation and classification algorithm**. Roughly 2000 conformations are constructed using ChainTweak and side-chains are added using RAPPER. The optimization is carried out until the fit-to-data converges (*R_free_*). In the final step, structures that collectively represent the data as well as the PDB structure are selected for classification via Lasso. EDM: Electron density map. *R *is a measure of agreement between the amplitudes of the structure factors calculated from a structure and those from the original diffraction data. *R_free _*is the corresponding cross-validation parameter, calculated on diffraction data not used in the structure optimization process [20].

Our method consists of two steps (Figure 1): a) construction of an ensemble representative of the observed data, and b) analysis of the variability in this ensemble using Lasso. The ensemble generation algorithm is independent of the classification of variability; the ensemble can be obtained from any other method. However, it is important to ensure that all the structures in the ensemble are of high-quality, and represent the data almost as well as the PDB structure (see Methods: *Ensemble Construction*). To formulate the classification problem using Lasso, we express the PDB structure as a linear combination of the members of the ensemble (each member is thus a feature). We fit the regression using EDMs obtained from the diffraction data and 3-D coordinates of individual members. Members of the ensemble that are noisy versions of the PDB structure, and hence more correlated with it, will be selected in this regression. The remaining structures are classified as truly variable (see Methods: *Analysis of variability and Electron Density Map*).

### Synthetic Data

**Figure 2:**
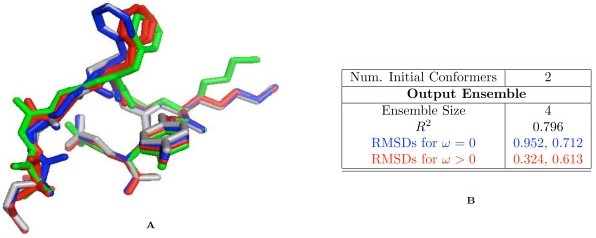
**Example of ensemble construction and classification**. A) PDB structure is shown in green, the second conformer in the synthetic crystal is in gray. The two structures classified by Lasso as variable are shown in blue and the two as variable due to noise, in red. B) Summary of the algorithm output using synthetic data. RMSD is calculated with respect to the PDB structure (green). Suitability of the linear model and statistical significance of the regression coefficients were evaluated using standard techniques (*R*^2 ^and *t-test*).

Our algorithm successfully models variability in a simulated crystal having two conformations, one the PDB structure (*conformer 1*) and the other constructed computationally (*conformer 2*) (Figure 2A; green and gray; RMSD = 0.989 Å). The second conformer was constructed using ChainTweak [21]; we randomly selected a conformation from a set of 100. Side chains were built using RAPPER and all atoms were assigned a B-factor of 30 *Å*^2^. Synthetic diffraction data were computed by averaging the simulated structure factors of the two conformers using the experimental resolution cutoffs [12,22].

Starting from an EDM of the simulated crystal, our algorithm generates structures similar to both the original structures (Figure 2A, B). Of the 13 structures output by the algorithm, 4 structures were non-redundant; remaining structures were almost identical to these 4 structures. Lasso regression on these 4 structures shows that the ensemble correctly identifies the heterogeneity in the original data; 2 structures have coefficients *ω *≈ 0 with regression done with EDM of *conformer 1 *(as per a *t-test; *colored blue in Figure 2A), corresponding to structures with true variability (see Methods: *Electron Density Map*). Moreover, the same conformations had statistically significant coefficients (*ω *> 0) in the regression with the EDM of *conformer 2*. Indeed, these conformations are closer to *conformer 2 *(RMSDs = 0.298, 0.128 Å) than the conformations classified as non-variable (RMSDs = 0.456, 0.765 Å). The algorithm thus appears to recover the heterogeneity in the data (Figure 2B).

#### Performance analysis

**Figure 3:**
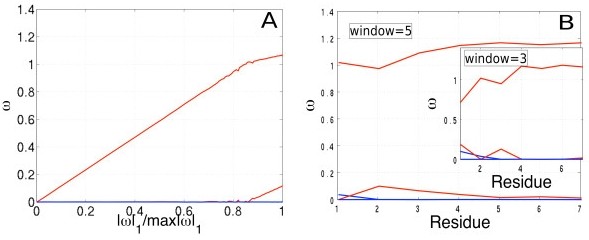
**Performance analysis**. A) Regularization path for the ensemble (|*ω*|_1 _→ 0 as *λ *→ ∞ towards left). B) Residue-level lasso with varying window sizes centered on each residue (*λ *= 10). The color code is the same as in Fig 2.

Our method is robust and consistent (Figure 3A, B). The consistency and accuracy of our method depends on the extent of correlation between the features (see Methods: *Analysis of variability*). Correlation between structures that are truly variable and ones variable due to noise, will result in different regularization penalties (*λ*) selecting very different structures, leading to highly variable regression weights [23,24]. Our simulations indicate that the features (i.e. conformations in the ensemble) are uncorrelated to a large extent (Figure 3A), indicated by the overall smooth trends for *ω *as we increase the regularization penalty *λ*. Increasing *λ *shrinks the individual weights of the features towards zero, thereby decreasing the ratio |*ω*|_1_/max|*ω*|_1_. We believe the overall smoothness of the regularization path may be due to the efficiency of the sampling algorithm- ChainTweak, which constructs highly diverse and uncorrelated conformations. In our simulations we find that, of the four structures in the ensemble, only one structure (red) is dominant for all regularization penalties. A second structure (red) is selected only at low *λ*'s (< 50; Figure 3A).

We find that our overall classifications are quite robust to the size of optimized grid region 'G' (see Methods: *Electron Density Map*). The average weight of a structure, calculated by averaging over all fragments, is consistent across varying fragment and window sizes; structures represented in red do indeed have the highest average weights and those in blue, negligible average weights (Figure 3B). One could vary 'G' in two ways: by splitting the chain into separate fragments and carrying out Lasso on each one, or by sliding a window centered around each residue and optimizing over each window. Our results on the fragment-based approach are identical to Figure 3B; we used fragment sizes of 1,2,4 and 8 (data not shown). For the second approach, we used sliding windows of sizes 3 and 5 centered on each residue (Figure 3B), and optimized over the bounding box enclosing the residues in the window.

#### Comparative analysis

**Figure 4:**
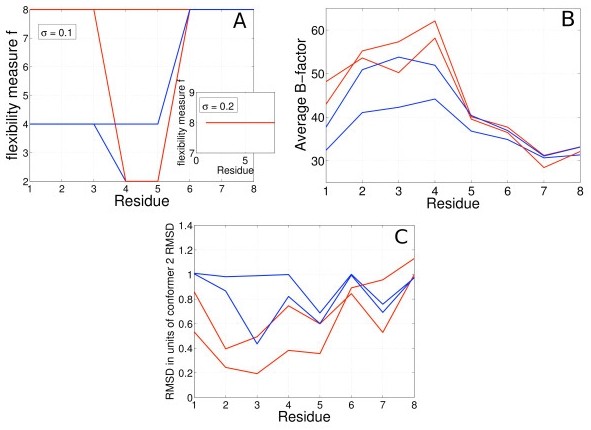
**Comparative analysis**. A) *Pflex *is sensitive to the parameter *σ*, producing false-positives at low values and false-negatives at higher values (inset). B) Average B-factors correctly identify the regions of variability, but cannot distinguish between true variability and variability due to noise. C) Choosing a RMSD cutoff for classification is difficult with noisy coordinates. The color code is the one used in Figures 2 and 3.

Lasso compares favorably to other methods in identifying true flexibility. The pairwise comparison method of Nigham et al. (*Pflex*) is sensitive to the standard deviation of added noise (*σ*). *Pflex *computes a flexibility measure, 'f', for each residue based on RMSD, *σ *and a threshold p-value. A lower f implies higher flexibility. We used the values suggested by Nigham et al. for *σ *(0.1 < = *σ *< = 0.2) and the threshold p-value (= 0.0001). *Pflex *tends to easily classify structures as variable at low levels of added noise (*σ *= 0.1, Figure 4A); three of the four structures in the ensemble are classified as variable. At higher noise levels it fails to classify any structure as truly variable, leading to false negatives; f remains at 8 for all residues for all structures in the ensemble (*σ *= 0.2, Figure 4A Inset). While B-factors can correctly identify the regions of high variability, they fail to distinguish between noise and true variability, as evidenced by the similar profiles (Figure 4B). RMSD (best-fit) provides some indication of the true variability, but the interpretation may be sensitive to noise levels. The extent of the initial variability in the crystal, represented by each structure in the ensemble can be analyzed by looking at normalized RMSD: RMSD from the PDB structure normalized by the RMSD of *conformer 2 *(from the PDB structure). A higher normalized RMSD implies the structure is closer to *conformer 2*, and a lower score implies it is closer to the PDB structure (Figure 4C). However, it is not clear what RMSD cutoff one should use in the presence of noise to robustly classify a structure as variable.

### Real Data

Our algorithm performs well on experimental diffraction data from 5 crystal structures across a range of resolutions (Table 1). We evaluated our models by comparing them with the best available single-conformer model (i.e., PDB). Analysis of data fits and variability amongst the models emphasizes the advantages of representing the data using multiple conformers. Even when our ensemble contains models differing by ~1 Å, we get an equivalent/improved fit to data:  is lower than or equal to the PDB *R_free_*. Our average improvements in *R_free _*are competitive with other approaches that construct multiple-conformer representations [7,9,12].

**Table 1:** Summary of the models obtained using real diffraction data.

PDB id	1ew4	1q4r	3di9	9ilb	1a3s
Resolution(*Å*)	1.4	1.9	2.0	2.3	2.8

No. Of reflections	22183	7578	22017	9535	5605

PDB R	0.206	0.187	0.244	0.156	0.176

PDB *R_free_*	0.229	0.245	0.264	0.193	0.236

	0.228	0.216	0.237	0.193	0.240

Ensemble Size	77	4	40	5	11

RMSD (*Å*)	0.792-1.13	0.678-0.859	0.728-1.085	0.805-1.413	0.826-1.238

PDB *R *and *R*_*free *_are calculated after 6 iterations of optimization in PHENIX [25]. These may differ from published values. "No. of reflections" gives the total number of experimental observations (i.e. intensity measurements).  measures the collective ability of the ensemble to represent the data.  is calculated in the same way as *R*_*free*_, except that amplitudes of structure factors averaged over the ensemble are used to calculate the residual [7]. Small RMSD ranges observed for the ensemble highlight the challenges of identifying true variability in real data.

Tests on real data show that multi-conformer models add the most value at low resolutions; at high resolutions (< 1.5*Å*) the ensemble is not able to significantly improve upon the fit-to-data (Table 1 PDB ). It is possible that the truly variable conformers themselves cluster into a small number of sets. This may be especially true for PDB structures 3di9 and 1ew4, where the greater number of observations might have a bearing on the larger size of the ensemble. Moreover, for low resolutions, it is interesting to note that most of the variability observed is due to noise - less than 8 alternate conformers are truly variable in most cases. This re-confirms the importance of analyzing the basis of variability, particularly in multi-conformer representations of low resolution data. Our method is suited for this analysis as the structures are selected robustly and the resulting sparsity can be physically interpreted.

**Figure 5:**
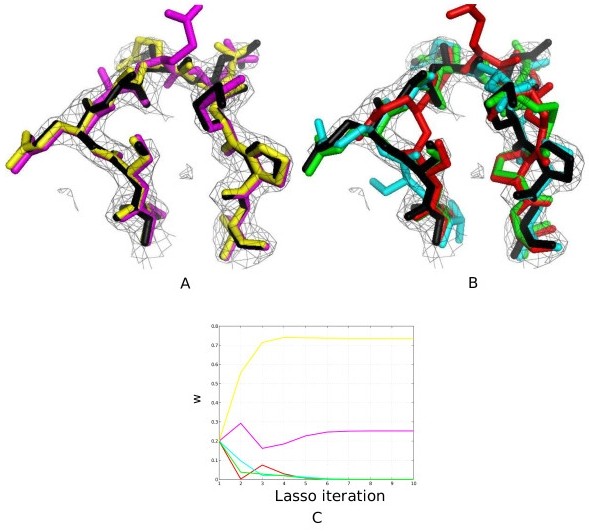
**Interpretation of ensembles on real data**. A) Lasso tests on 9ilb:124-132 classifies 2 structures as non-variable (pink, yellow). B) For the same loop, structures classified as truly variable (red, cyan, green) deviate more from the PDB structure (black). C) Trajectory of the solution can give qualitative knowledge of the landscape in the vicinity of the native structure. All density maps are contoured at 1.5*σ *for clarity. Figures were generated using PyMol [36].

We observe that Lasso can classify variability effectively for most cases; structures classified as variable appear to differ more than those classified as non-variable (Figure 5A, B). Since we use an iterative method to solve the regression problem, interpretation of variability in the ensemble can be further analyzed by looking at the solution trajectory for Lasso (*ω *vs. Lasso iteration; Figure 5C). The trajectory can help give a qualitative picture of the landscape near the native conformation: structures whose coefficients go to zero faster are farther away from the native structure.

**Figure 6:**
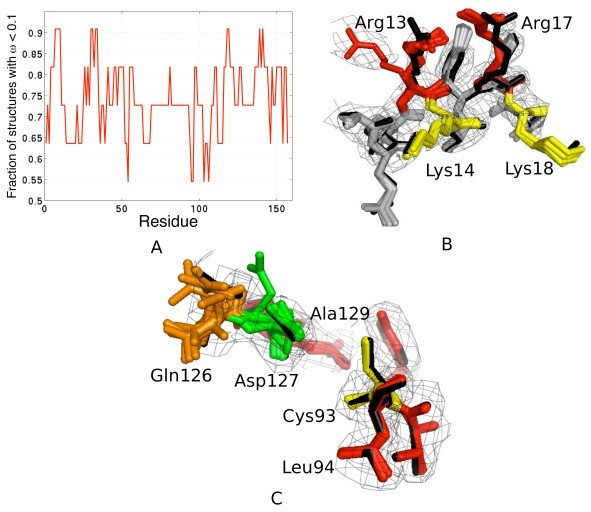
**Flexibility analysis of the 1a3s ensemble**. A) Residue level Lasso with a window size of 5 reveals four fragments (peaks) of potential interest: 6-15, 30-40, 115-120 and135-142. B) The N-terminal region (12-20) of 1a3s. Multiple rotamers of R13 (left, red) might affect the interaction surface consisting of R18 (red), K14 and K18 (yellow), thus influencing Ubc9's N-terminus specificity. C) Variability around the catalytic site Cys93 (yellow). Residues Gln126 (brown) and Asp127 (green) have been identified through mutagenesis experiments as critical for Ubc9's interaction with a substrate. The black structure represents PDB coordinates.

We then asked the question: "is there any biological insight from the ensemble that can help us in understanding protein function ?" To this end, our results on the crystal structure of the human ubiquitin-conjugating enzyme (Ubc9, pdbid: 1a3s) give some interesting anecdotal evidence [26]. Using a window-size of 5 centered on each residue, we applied Lasso to identify the most variable regions for 1a3s (11 structures; Figure 6A). Four fragments turn out to be highly variable: the N-terminal helix (6-20), 30-40, 115-120 and C-terminus residues 135-145. This is in good agreement with NMR experiments, which reveal that Leu6, Ala10, Arg13, Arg17, Leu38, Leu119, Gln126, Asp127, Ala129, Glu132, Ile136 and Asn140 are amongst the most flexible residues in an otherwise rigid structure [27,28]. These residues overlap with our predictions of the true variable regions (Figure 6A). Our method is thus able to identify physically relevant variabilities.

Additionally, it is known that the N-terminus is important for Ubc9's specificity for SUMO rather than ubiquitin [27,28]. However, the molecular mechanisms responsible for substrate identification and interaction are not well understood [29]. Tatham et al. (2003) conducted site-directed mutagenesis experiments on Ubc9 to discover that mutations R13A/K14A and R17A/K18A disrupted Ubc9's interaction with SUMO-1. More recently, through a crystal structure of the Ubc9-SUMO-1 complex, R13 and R17 have been observed to be involved in key non-covalent interactions with SUMO-1 [30]. A closer look at the heterogeneity modeled by our method suggests two possible conformational states for R13 (Figure 6B). Proximity of the two arginines at positions 13 and 17 indicate that such conformational changes might influence the binding interface with an E1-ubiquitin conjugate [28,30]. Finally, we looked at the dynamics captured by the ensemble near the active site for substrate recognition Cys93. We find that Gln126 and Asp127 have been modeled in multiple conformations by our algorithm (Figure 6C). Interestingly, these residues have been shown to be highly flexible and possibly important for substrate recognition through mutagenesis experiments [31]. Further detailed analysis in light of our results could give some insight into the molecular mechanisms underlying such specific interactions.

## Conclusions

We have introduced a novel technique for analyzing conformational changes that may be present in a real protein crystal. Our method first constructs a high-quality, diverse ensemble of structures respresentative of the crystallographic data. We then use a sparse estimation algorithm (Lasso) to distinguish structures that are genuinely variable from those that appear variable due to noise.

Unlike previous approaches, our method involves the estimation of variability by operating in the EDM space rather than in the 3-D coordinate space. This allows us to avoid the errors that are implicitly introduced in inferring the 3-D coordinates from the EDM. In particular, our method is able to effectively deal with correlated motions, without assuming independent and identically distributed (i.i.d) noise - a key assumption in earlier approaches [11]. Tests on real data show that the algorithm is able to capture physically relevant conformational changes, even for low resolution structures where the amount of noise is significant. Another advantage of operating in EDM-space is that our current technique is independent of any structure inference packages, and can be integrated to improve structure inference at an earlier stage in the structure-building process (e.g., from an initial experimental EDM). We believe that this approach is particularly useful in inferring/analyzing low-resolution structures. A common criticism of ensemble modeling approaches at low-resolutions is that they over-fit the data [9,12]. In contrast, our use of Lasso enables us to identify and discard structures that are variable only due to noise, permitting simultaneous optimization of the ensemble against the data without significant over-fitting risk. This, in turn, should improve automated structure determination at low resolutions where ambiguous EDMs often lead to error-prone single conformer models [9,12].

A key contribution of this paper is the Lasso-based statistical test to distinguish variability due to noise from that due to true heterogeneity. We believe that the general approach we have introduced - to evaluate noise using the entire ensemble, rather than on a per-atom pairwise basis - may be of value in other ensemble based analyses also. Lasso's performance as a statistical test here could be further improved by using kernel-based algorithms that can effectively deal with correlations and non-linear generative models [32].

Correctly estimating the true variability in a protein's structure is crucial. Our results indicate that the magnitude of variations within the ensemble could give misleading results for structural analysis, especially with single conformer models. Furthermore, true, localized variability could have a significant impact on solvent accessibilities, secondary structure prediction, estimation of electrostatic and potential energies, and template-based homology modeling techniques [8]. Accurate estimate of these quantities, in turn, is crucial to understanding the biochemical and functional characteristics of a protein.

## Methods

### Ensemble Construction

To obtain a diverse, high-quality ensemble representing the X-ray diffraction data, we seed a single-conformer maximum likelihood optimization procedure (e.g., PHENIX) with a diverse set of conformations [25]. We assume that realistic conformations explaining the crystallographic data will be within a limited RMSD distance of the published PDB structure; this follows similar assumptions in previous work [7,10]. However, hinge motion, if present in a single crystal specimen, can also be detected by sampling in a larger conformational space around the PDB structure. Starting from the backbone coordinates in the PDB, we construct alternate backbone conformations within 2Å RMSD using ChainTweak, a state-of-the-art inverse-kinematics based neighborhood-sampling algorithm [21]. ChainTweak can, in principle, exhaustively sample from the neighborhood of a conformation; leading to a highly variable and diverse ensemble. For each backbone, we assign side-chains using RAPPER [7], based on their fit to the Electron Density Map (EDM). We tried sampling from higher RMSD neighborhoods around the PDB structure, but RAPPER often fails to find a rotamer-assignment compatible with the EDM for conformations greater than 2Å RMSD from the PDB backbone. The final ensemble is obtained by subsequent optimization using PHENIX and filtering based on fit-to-data, measured using a cross-validation parameter *R_free_*; lower *R_free _*implies better fit-to-data. The final ensemble consists of structures that are of high quality and collectively represent the data, as well as the PDB structure (Figure 1).

### Analysis of Variability using Lasso

Given an ensemble of conformations, our goal in this section is to identify the subset of conformations whose variation from a given base conformation is most likely due to only noise in the experimental data. The remaining conformations can then be interpreted as demonstrating true variability compared to the base conformation. The choice of a base conformation here is arbitrary; a natural choice for it is the PDB structure, since one is often interested in conformational variability not captured by the published PDB structure. To achieve this goal, we formulate a Lasso regression problem: we express the base conformation as a linear combination of the ensemble members (each such conformation is thus a feature); we use experimental data (i.e. diffraction data) to fit this regression. As part of the Lasso framework for feature selection, we assign (unknown) weights to each feature. The key strength of Lasso is that it is likely to make the weights for irrelevant features exactly zero, clearly identifying them. The intuition here is that structures sampled from a Gaussian distribution (i.e. modeled by B-factors) about the PDB structure should be more predictive of the PDB structure than structures displaying true variability. The former structures will be assigned a non-zero weight during Lasso and can then be classified as not displaying true structural variability, since they are adequately represented by the PDB structure and do not represent biologically relevant long time-scale motion.

Lasso regression is often an effective technique for shrinkage and feature selection in cases where feature selection must be performed with noisy, limited data [16,32,33]. The loss function of Lasso regression is defined as:(1)

where *x_ip _*denotes the *p*th predictor (feature) in the *i*th data point, *y_i _*denotes the value of the response for this data point, and *β_p _*denotes the regression coefficient of the *p*th feature. The *l*_1 _regularizer leads to a sparse solution in the feature space, which means that regression coefficients for the most irrelevant and redundant features shrink to zero. Interestingly, recent theoretical work recovers Lasso as a formulation of a linear robust regression problem under feature-wise uncorrelated and norm-bounded noise [33]. The authors suggest that such problems are of interest when values of the features are obtained with noisy pre-processing steps, and the magnitudes of such noises are bounded.

We exploit this parallel in our formulation, where we compute each feature (i.e. each structure in the ensemble) by optimizing against the observed data. The PDB structure is the observed quantity, and the individually optimized structures in the ensemble are our noisy predictor features. A sparse solution in the *β *space will then represent structures which are variable due to noise (*β_p _*> 0), thus decomposing the variability observed in the ensemble. To get the regularization penalty *λ*, we follow suggestions based on other applications of Lasso and use cross-validation [16,24].

### Electron Density Map

Lasso regression can be performed either in the coordinate space or the electron density space (EDM). In contrast to previous approaches, which use coordinate based methods for pairwise structure comparison, we have designed the test using EDMs, since the former cannot distinguish between model errors and genuine structural outliers [17]. EDMs are obtained by taking an inverse-fourier transform of the observed diffraction data, which are appropriately scaled using B-factors [22]. Another advantage of using an EDM is that it directly includes the B-factors of the models, and hence can also inherently deal with isotropic or anisotropic B-factors. This circumvents the problem of estimating actual uncertainty from B-factors, which is often a challenge for coordinate based methods. The simple regression test quantifies the relevance of each structure in the ensemble to the Gaussian distribution around the PDB (as given by the B-factors).

As part of our Lasso formulation, we assume as the observed variable, the EDM computed from the PDB structure. The predictor variables, or features, are EDMs of structures in the ensemble. The electron density at a point 'g' on a grid describing the observed EDM , is then modeled as a linear combination of electron densities at the point 'g' of the predictor EDMs . We assume that the observed electron density is noisy with respect to our generative model and model this using a normally distributed noise component *ε_g_*. We then minimize the Lasso loss function:(2)(3)

Here, *ω*_*i *_are the regression coefficients. The structures for which *ω*_*i *_approaches zero are the ones most irrelevant compared to the PDB, and hence exhibit true variability. To optimize over a fragment (e.g., one residue), 'g' is restricted to the bounding box for the fragment.

All EDMs are constructed using Clipper [34], and are described on the same unit cell with the same symmetry as that of the PDB structure. The optimization was carried out using the non-linear optimization library IPOPT, which uses an interior point method, combined with an efficient line-search procedure, to minimize the non-linear objective function [35].

## Competing interests

The authors declare that they have no competing interests.

## Authors' contributions

RS, RH and BB identified the problem. RH implemented the algorithms and collected data. RH, RS and BB analyzed the data and wrote the paper. All the authors have read and approved the final manuscript.
